# Supplementary material for: The Superiority of Bacillus megaterium over Escherichia coli as a Recombinant Bacterial Host for Hyaluronic Acid Production
Source: Microorganisms. 2022 Nov 28;10(12):2347. doi: 10.3390/microorganisms10122347 (PMC9787986; doi:10.3390/microorganisms10122347)
Supplement: Supplementary file 1 [file microorganisms-10-02347-s001.zip › microorganisms-2041033-supplementary.pdf]

Table S1. Selection of HA producing *E.coli* Colonies.

Strain	Plate one	Plate two	Plate three
pMM*hasA* Rosetta2	LB+glucose1%+chloramphenicol34ug/ml	LB+glucose1% +0.5%Xylose	LB+glucose1%+0.5%Xylose + Ampicillin100ug/ml
pP$_{T7}$ *has ABC LysY/Iq*	LB+glucose1%	LB+glucose1%+0.5 mM IPTG	LB+glucose1%+1mM IPTG+ Ampicillin100ug/ml
pPT$_7$*hasABC* Rosetta2 DE3pLysS	LB+glucose1%+ chloramphenicol 34ug/ml	LB+glucose1%+0.5 mM IPTG + chloramphenicol34ug/ml	LB+glucose1%+ Chlorampheincol 34ug/ml +1mM IPTG+ Ampicillin100ug/ml
pPT$_7$*hasABC* Rosetta B-gammiDE3pLysS	LB+glucose1%+ chloramphenicol34 ug/ml	LB+glucose1%+0.5 mMIPTG + chloramphenicol34ug/ml	LB+glucose1% + Chlorampheincol 34ug/ml +1mM IPTG+ Ampicillin100ug/ml
pPT$_7$*hasABC*DE Rosetta B-gammiDE3pLysS	LB+glucose1%+ chloramphenicol34 ug/ml	LB+glucose1%+0.5 mMIPTG + chloramphenicol34ug/ml	LB+glucose1% + Chlorampheincol 34ug/ml +1mM IPTG+ Ampicillin100ug/ml

Table S2. List of oligonucleotides.

Primers Name	Sequence (Restriction sites)	Purpose
Forward *hasA* pJet	ATCGGATCCTGAGGAGACACAACATGAGAACATTAAAAAACCT (*Bam*HI).	Cloning in pJet and pMM1522
Reverse *hasA* pJet	AGAATTGAGGCTCTTATAATTTTTTACGTGT (*Sph*I).	Cloning in pJet and pMM1522
Reverse *hasAB* pJet	TTCTGAGAGGCTCTAGTCTCTTCCAAAGAC (*Sph*I).	Cloning in pJet and pP$_{T7}$
Reverse *hasABC* pJet	TATCGGCATGCTTACTGGGGCTGATC (*Sph*I).	Cloning in pJet and pP$_{T7}$
Forward *hasABCDE* pP$_{T7}$	ATAATCAGATCTGAGGAGACACAACATGAGAACATTAAAAAACCT (*Bgl*II).	Cloning in pP$_{T7}$
Reverse *hasABC* pP$_{T7}$	ATAATCAGATCTTTACTGGGGCTGATC (*Bgl*II).	Cloning in pP$_{T7}$
Reverse *hasABCDE* pP$_{T7}$	ACTGATCCCGGGTTACAAGCGTGC (*Xma*I).	Cloning in pP$_{T7}$

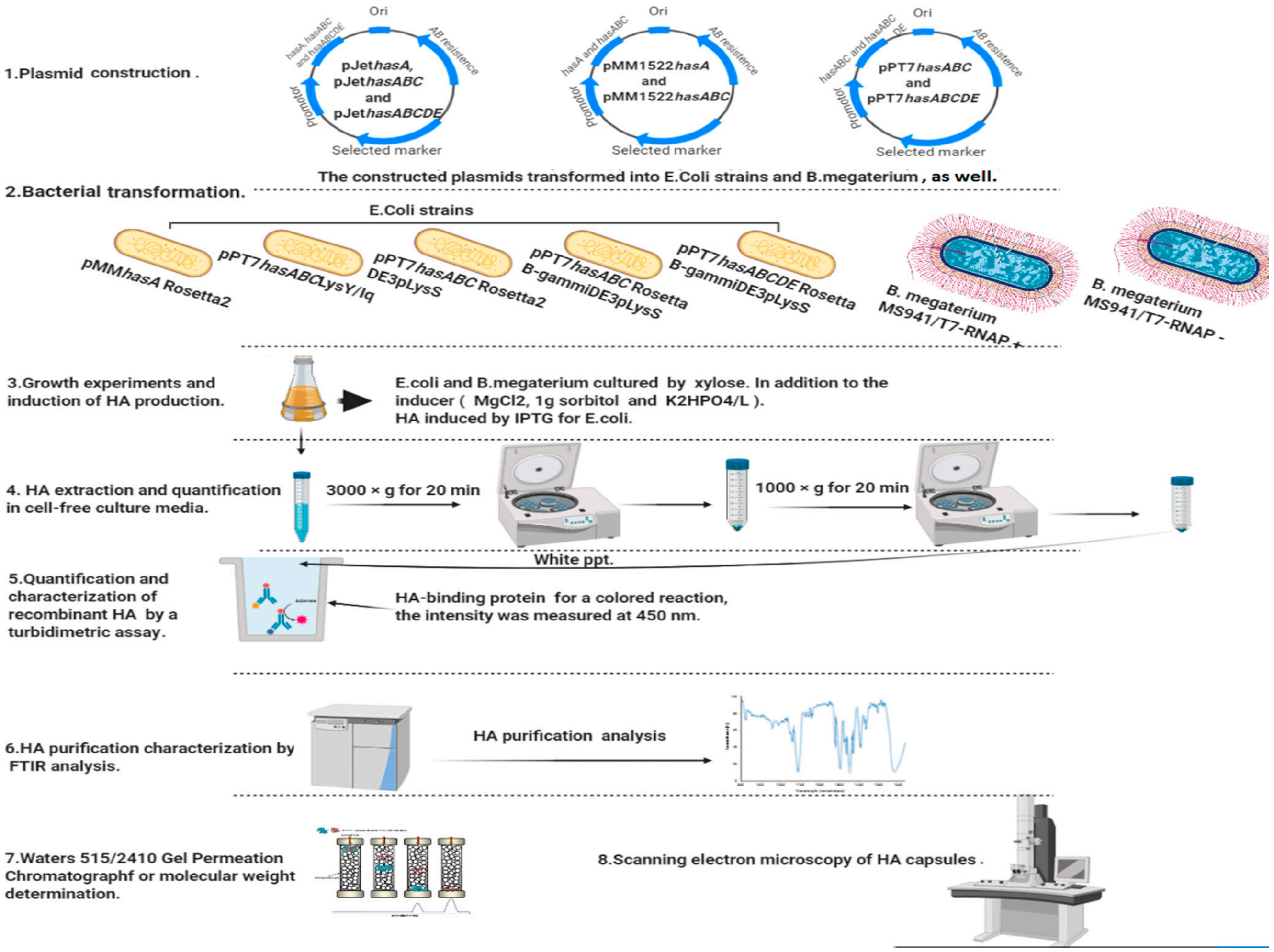

Figure S1. The graphical abstract of Materials and Methods.

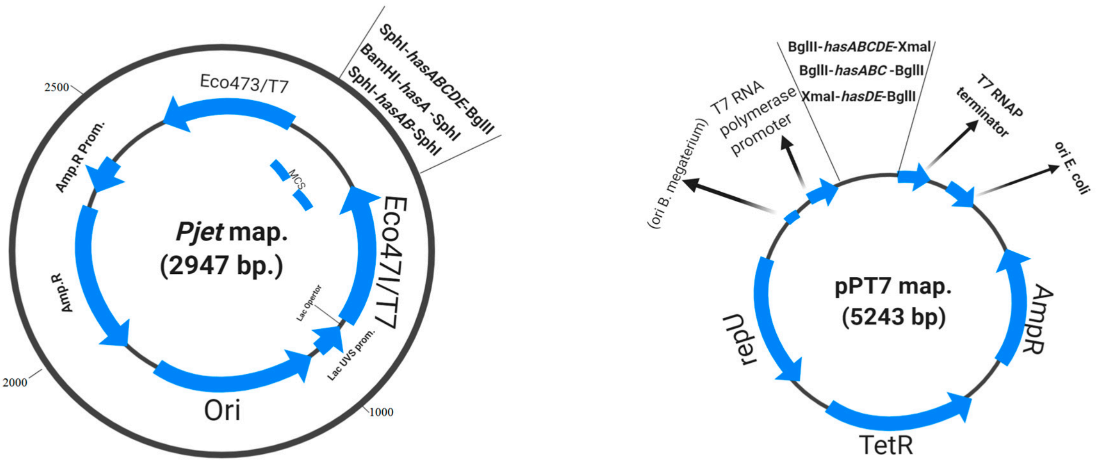

Figure S2. Plasmid construction of *hasaABC/DE* cloned into Pjet and pP$_{T7}$

Table S3. Selection of HA producing *Bacillus megaterium* MS941Colonies

Strain	Plate One	Plate Two	Plate Three
pMM1522*hasA* in *B.megaterium* MS941	LB + glucose1 %	LB + glucose1 % + 0.5 % Xylose	LB + glucose1 % + 0.5 %Xylose + tetracycline 4 ug/ml
pP$_{T7}$ *has ABC* in *B. megaterium* MS941pretransformed with T7RNAP.	LB + glucose1 % + chloramphenicol 3.4 ug/ml	LB + glucose1 % + chloramphenicol 3.4 ug/ml + 0.5 % Xylose	LB + glucose 1 %+ chloramphenicol 3.4 ug/ml + 0.5 % Xylose + tetracycline 4 ug/ml
pP$_{T7}$*has ABCDE* in *B. megaterium* MS941pretransformed with T7RNAP	LB + glucose 1 % + chloramphenicol 3.4 ug/ml	LB + glucose 1 % + chloramphenicol 3.4 ug/ml + 0.5 % Xylose	LB + glucose 1 % + chloramphenicol 3.4 ug/ml + 0.5 % Xylose + tetracycline 4 ug/ml

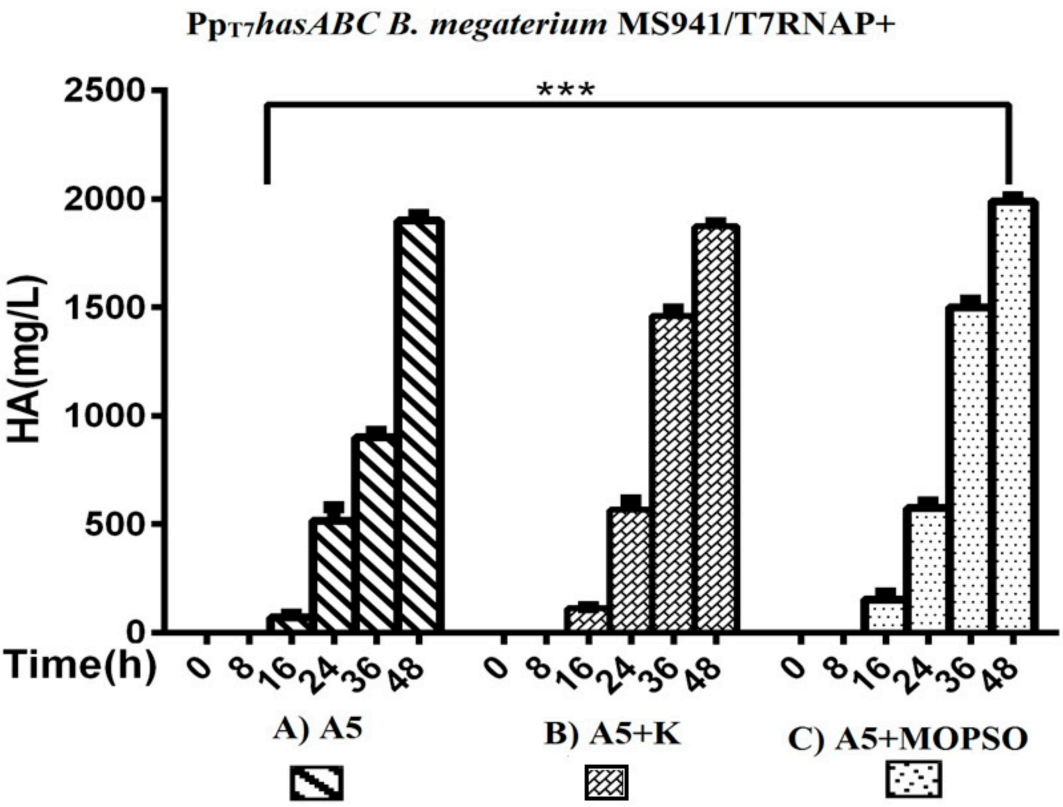

Figure S3. HA production *by* pP$_{T7}$*hasABCDE B. megaterium* MS941 pre-transformed with T7RNAP in (**A**) A5, (**B**) A5 + K and (**C**) A5 + MOPSO, (***): $P < 0.0001$.

Table S4. FTIR peaks; reference HA-standard FTIR, and HA produced by pP_{T7}*hasABCDE E. coli* Rosetta-gami B pLysS and pP_{T7}*hasABCDE B***.**

**Wave length (cm^{-1})**	**Functional group**
3600-3604	confirms the presence of OH stretching
2897.89-2936.59	C-H stretching
1612.79-1653.02	Presence of amide II group
1410.64-1414.68	presence of C-O group with C=O combination,
1042.30-1058.73	C-O-C stretching
612.32-555.39	C-O-C stretching
3600-3605	confirms the presence of OH stretching
2897.89-2937.73	C-H stretching
1612.79-1650.02	Presence of amide II group
1410.64-1425.93	presence of C-O group with C=O combination,
1042.30-1058.49	C-O-C stretching
612.32-520.39	C-O-C stretching
